# Cross sectional analysis of gut microbiota of ALS patients with and without percutaneous endoscopic gastrostomy

**DOI:** 10.3389/fmicb.2026.1842792

**Published:** 2026-08-05

**Authors:** Jose Enrique de la Rubia Ortí, Guillermo Bargues-Navarro, Sandra Sancho-Castillo, Jesús Privado, María Benlloch García, Claudia Emmanuela Sanchis Sanchis, Laura Garcia Martinez, María Cuerda-Ballester, Palmira Martínez Bolós, Francisco Jose Roig

**Affiliations:** 1 Department of Basic Biomedical Sciences, Catholic University of Valencia, Valencia, Spain; 2 Doctoral School, Catholic University of Valencia, Valencia, Spain; 3 Health Research Institute (IIS) La Fe, La Fe Polytechnic and University Hospital, Valencia, Spain; 4 Department of Pathology, Catholic University of Valencia, Valencia, Spain; 5 Department of Methodology of Behavioral Sciences, Universidad Complutense de Madrid, Madrid, Spain; 6 Departament of Nursing, Catholic University of Valencia San Vicente Mártir, Valencia, Spain; 7 Facultad de Ciencias de la Salud, Universidad San Jorge, Zaragoza, Spain; 8 Biamics, Servicios de Bioinformática SL, Zaragoza, Spain

**Keywords:** amyotrophic lateral sclerosis, bacterial diversity, gut microbiota, metagenomics, nutritional assessment, percutaneous endoscopic gastrostomy

## Abstract

**Introduction:**

This cross-sectional study investigated the differences in gut microbiota in patients with Amyotrophic Lateral Sclerosis (ALS) with and without percutaneous endoscopic gastrostomy (PEG), exploring their cross-sectional associations with nutritional intake.

**Methods:**

Use of shotgun metagenomics and dietary assessments.

**Results:**

We identified significant taxonomic shifts and changes in diversity across groups. PEG patients exhibited reduced abundance of short-chain fatty acids (SCFAs)- producing genera, such as *Faecalibacterium* and *Lachnospira*, suggesting a dysbiotic profile; the Firmicutes/Bacteroidetes ratio was also lower in PEG patients but is reported as a descriptive indicator only. Correlations between specific bacterial taxa and nutrient intake, highlight the potential role of the gut microbiota in ALS pathophysiology. These findings describe cross-sectional differences in microbial composition associated with nutritional status and feeding route.

**Discussion:**

Our results provide a foundation for microbiome-targeted interventions in the management of ALS, although findings related to PEG should be interpreted as exploratory given the limited sample size. Furthermore, all comparisons involving the external control group (BioProject PRJNA961076) must be interpreted with caution due to potential batch effects from differences in sample collection, DNA extraction kits, and sequencing platforms.

## 1 Introduction

Amyotrophic Lateral Sclerosis (ALS) is a progressive neurodegenerative disorder that primarily targets motor neurons in the brain and spinal cord. It may present with bulbar onset, when motor neurons in the brainstem are first affected, or with spinal onset, when the disease predominantly involves motor neurons in the spinal cord (Gordon, 2013).

ALS displays marked clinical heterogeneity in phenotype, rate of functional decline, nutritional status, and survival, suggesting that factors beyond primary motor neuron degeneration may contribute to disease expression (Chakraborty et al., 2026; Ayala et al., 2025; Gautam et al., 2021).

Dysphagia is one of the most significant challenges faced by patients with ALS, affecting up to 70% of individuals with the disease (Burkhardt et al., 2017). Dysphagia impairs nutritional intake and increases the risk of aspiration pneumonia. Percutaneous Endoscopic Gastrostomy (PEG) is often recommended to address these challenges (Mazzini et al., 1995).

PEG is typically recommended when patients experience significant weight loss, malnutrition, dehydration, or when oral intake becomes excessively time-consuming and fatiguing (Cui et al., 2018). Notably, patients with ALS often exhibit altered nutritional habits and inadequate dietary intake, which are associated with accelerated disease progression and poorer clinical outcomes (D'Antona et al., 2021; Dupuis et al., 2011; Rosenfeld and Ellis, 2008).

The gut microbiota plays a crucial role in maintaining the overall health. When an imbalance -dysbiosis- occurs, negative health outcomes can manifest (Afzaal et al., 2022). Gut dysbiosis can result from various intrinsic and extrinsic factors, such as diet, stress, drug or antibiotic use, infections, and chronic diseases (Lee et al., 2024a; Safarchi et al., 2025).

Recent human studies using shotgun metagenomic sequencing have reported gut microbiota alterations in ALS patients compared with healthy controls, including changes in taxonomic composition and microbial gene content (Gautam et al., 2025a; Guo et al., 2024).

In the context of ALS, recent studies have highlighted the potential role of the gut microbiota in disease progression. Patients with ALS often exhibit significant gut dysbiosis, characterized by an altered Firmicutes/Bacteroidetes ratio, which is associated with increased microbial translocation to the blood and more severe symptoms (Kim et al., 2022). Gut-derived neurotoxins and excitotoxins produced by dysbiotic microbiota may contribute to the neurodegenerative processes observed in ALS (Lee et al., 2024b).

However, the majority of available evidence is cross-sectional and heterogeneous in design, precluding causal inference and requiring cautious interpretation (Chakraborty et al., 2026; Gautam et al., 2026).

Despite growing evidence linking gut dysbiosis to ALS progression, the specific impact of enteral nutrition via PEG on gut microbiota composition remains poorly characterized. Unlike oral feeding, PEG bypasses mastication and oropharyngeal modulation of the gut microbiome, delivers nutrients directly to the stomach in a standardized formulation, and is typically introduced at advanced disease stages when dysbiosis may already be established. Whether PEG further disrupts microbial composition—or partially stabilizes it through consistent nutrient delivery—remains an open question with direct clinical relevance for the nutritional management of ALS patients.

Accordingly, the present study uses shotgun metagenomic sequencing to explore cross-sectional associations between gut microbiota composition and PEG status in ALS patients, with an explicitly exploratory and hypothesis-generating intent.

## 2 Materials and methods

### 2.1 Participants data collection

A total of 59 patients with ALS were evaluated, of whom five had a PEG, requiring nutrients to be administered directly into the stomach. As a control group, 15 metagenomic samples from healthy individuals were retrieved from Bioproject PRJNA961076.

### 2.2 Procedure

A cross-sectional selective sampling approach was used. Participants were recruited based on specific characteristics pertinent to the study objectives. The principal ALS associations across Spain were contacted to assemble the sample. The project was presented to the coordinators, who subsequently relayed the information to the patients at each affiliated center. Individuals who chose to participate voluntarily were enrolled.

The inclusion criteria comprised male participants aged over 18 years; non-fertile female participants over 50 years old, or females between 18 and 50 who were not intending to become pregnant; patients with a confirmed ALS diagnosis who had been symptomatic for a minimum of 6 months prior to enrollment; patients under treatment with Riluzole; and individuals who provided written informed consent to participate.

The exclusion criteria were as follows: presence of a tracheostomy; use of invasive or noninvasive ventilation involving positive pressure; concurrent participation in another clinical trial or participation within the previous 4 weeks; diagnosis of dementia; history of substance (alcohol or drug) abuse; positive status for hepatitis B, hepatitis C, or HIV; renal impairment characterized by creatinine levels twice the upper limit of normal within 30 days prior to inclusion; or liver dysfunction, defined as ALT and AST values exceeding three times the normal range within the same timeframe.

After applying these criteria, the final sample included 74 patients diagnosed with either bulbar or spinal onset ALS.

### 2.3 DNA extraction, sequencing and quantity control of reads

Fecal samples were collected from participants at their homes within 24 h before their hospital visit. Participants received the OMNIgene-GUT collection kit (OMR-200; DNA Genotek, Ottawa, Canada) in advance, along with detailed instructions on collecting and storing fecal samples. They were instructed to transfer a portion of their feces into the kit’s container, which contained a stabilizing solution and did not require refrigeration. Microbial genomic DNA was extracted from fecal samples within 1 month using the DNeasy PowerSoil Pro Kit (Qiagen, Hilden, Germany), following the manufacturer’s instructions.

Samples were sequenced using shotgun metagenomics on the Illumina NovaSeq6000 platform with an S4 flow cell (2 × 150 bp). DNA was extracted using the Qiagen DNeasy PowerSoil Pro kit, and libraries were prepared using the Illumina DNA Prep kit. Demultiplexing was performed using the bcl2fastq tool (version 2.20.0.422), allowing a maximum of one mismatch in the barcode. Preprocessing involved using TrimGalore (Krueger et al., 2016) to remove low-quality reads (quality score <Q20), short reads (<75 bp), and reads with more than two ambiguous nucleotides. Contaminant DNA was identified and removed using Bowtie2 (Langmead and Salzberg, 2012) with the -sensitive-local parameter, targeting phi X 174 Illumina spike-in and human-associated reads (hg19). This process resulted in standard forward, reverse, and unpaired read output files for each metagenome. Shotgun metagenomic sequencing enables taxonomic profiling and annotation of microbial gene content; however, functional information derived from these data was interpreted strictly as potential functional capacity and not as direct measures of microbial activity. The data can be found in accession number PRJEB90512.

Control samples were extracted from bioproject PRJNA961076 from Intestinal metagenome of healthy patients, samples SRR24315719, SRR24315720, SRR24315723, SRR24315724, SRR24315743, SRR24315746, SRR24315748, SRR24315750, SRR24315751, SRR24315753, SRR24315757, SRR24315759, SRR24315761, SRR24315763 and SRR24315765.

### 2.4 Technical procedure

Any analysis aimed at comparing groups or evaluating cross-sectional clinical associations was performed using the phenotypic data gathered during the study.

### 2.5 Taxonomic profiling

The microbial taxonomic composition of all samples was analyzed using MetaPhlAn4 (Blanco-Míguez et al., 2023) and the mpa_vJan21_CHOCOPhlAnSGB_202103 database (available at http://cmprod1.cibio.unitn.it/biobakery4/metaphlan_databases/) with default parameters. Quality-controlled mapping results were used by MetaPhlAn4 to calculate the coverage of each marker gene and determine the relative abundance of each taxonomic clade. Estimated taxon counts were obtained by scaling MetaPhlAn relative abundances by the total number of sequencing reads per sample, and the resulting pseudo-counts were used for the different analysis.

Prior to any analysis, taxa were filtered based on their prevalence across samples: a taxon was considered valid and retained for analysis if it was detected in at least 70% of the samples in at least one of the three groups (ALS, PEG or Controls). This criterion ensures that reported results reflect taxa with consistent representation across subjects, minimizing the influence of sporadic or low-prevalence features. For reporting purposes in the results section, mean taxon abundances are expressed as the percentage contribution of each taxon to the total estimated counts within each group, calculated as the mean estimated count of each taxon divided by the total mean estimated counts within the group.

### 2.6 Statistical analysis

#### 2.6.1 Statistical analysis

All statistical analyses were performed using a custom pipeline combining Python 3 and R. Input data consisted of estimated taxon counts obtained from Metaphlan analysis.

Differential abundance analysis was performed for each pairwise group comparison using three complementary methods. (i) ANCOMBC2 (Lin and Peddada, 2024), implemented in the R package *ANCOMBC*, applied to count matrices wrapped in TreeSummarizedExperiment objects, with BH correction for multiple testing and no additional prevalence filtering, as filtering had been applied upstream. (ii) ALDEx2 (Fernandes et al., 2014), with 128 Monte Carlo Dirichlet instances (mc.samples = 128), using the Wilcoxon test (wi.ep) and its BH-adjusted q-value (wi.eBH) as significance metric. (iii) A Centered Log-Ratio linear model (CLR-LM) (Gloor et al., 2017), in which counts were CLR-transformed after addition of a pseudocount of 0.5 and a linear model was fitted per taxon with BH correction. A global random seed (42) was applied across all methods to ensure reproducibility. A taxon was considered significantly differentially abundant when identified as significant (*q* < 0.05) by at least two of the three methods.

Isometric log-ratio (ILR) analysis (Egozcue et al., 2003) was performed per pairwise comparison using the *compositions* R package. The ILR basis was computed from the full taxon matrix and a linear model was fitted per ILR coordinate, with BH correction applied to the resulting *p*-values.

Descriptive statistics and correlation analyses were computed in Python. For each pairwise group comparison, mean, median and standard deviation were calculated per taxon per group directly on the estimated counts. Spearman rank correlations were computed between the abundance of each taxon and the total count per sample within each group, as a measure of the association between individual taxon abundance and overall microbial load. For correlation analyses, raw Spearman p-values are reported given the exploratory nature of these analyses and the limited sample size involved.

### 2.7 Data visualization

For graphical representation and all analyses, custom scripts written in Python were used, leveraging well-known libraries such as Seaborn, Matplotlib, and Scikit-learn. These libraries provide robust tools for creating detailed and informative visualizations, ensuring reproducibility and flexibility in the data analysis process. The use of these advanced visualization and analysis techniques allowed for a comprehensive and detailed examination of the data, thereby enhancing the robustness and reliability of our findings.

*Taxonomic Diversity*, the representation of taxonomic diversity, and Krona analysis were conducted based on the identified lineages for the visualization of taxonomic data, allowing for an intuitive exploration of bacterial composition and hierarchical relationships within the metagenomic data. This method illustrates the distribution of taxonomy across different samples, providing insights into the diversity and composition of microbial communities.

The use of these advanced visualization and analysis techniques allowed for a comprehensive and detailed examination of the data, enhancing the robustness and reliability of our findings.

### 2.8 Diversity index

To evaluate microbial diversity within the samples, several alpha diversity indices were calculated using the R package vegan (Oksanen et al., 2022), including the Shannon index (Shannon, 1948), Simpson index (Simpson, 1949), observed richness, Chao1 richness estimator (Chao, 1984) and Abundance-based Coverage Estimator (ACE) (Chao and Lee, 1992). Between-group differences in alpha diversity were assessed using the Wilcoxon-Mann–Whitney test for two-group comparisons or the Kruskal–Wallis test for three or more groups.

For beta diversity, Aitchison distance was computed on CLR-transformed counts (Aitchison, 1986) after addition of a pseudocount of 0.5. Between-group differences in community composition were tested using PERMANOVA (adonis2, 999 permutations; Anderson, 2001), and homogeneity of multivariate dispersions was evaluated using betadisper with permutation testing, both implemented in vegan (Oksanen et al., 2022).

### 2.9 Diet and nutrition

A nutritional study was conducted to thoroughly assess the patients’ dietary habits during the months prior to the study. Nutritionists conducted the monitoring. The study analyzed the daily intake of micro- and macronutrients by the patients up to the point of data collection and fecal sample collection.

#### 2.9.1 Dietary and nutritional history and data collection

To initially assess the patients’ dietary intake, 24-h dietary records were collected over a seven-day period (Ortega et al., 2015), along with a Food Frequency Questionnaire (Trinidad Rodríguez et al., 2008).

These tools provided detailed information on the frequency of consumption of various food groups, including dairy, vegetables, fruits, juices, nuts, meats, fish, seafood, eggs, tubers, rice, legumes, pasta, processed meats, snacks, pastries, cookies, chocolate, sugary drinks, and both fermented and distilled alcoholic beverages. The seven-day period allowed for a representative snapshot of participants’ usual eating habits, minimizing the bias that could arise from analyzing a single day (Ortega et al., 2015).

The 24-h record consisted of a self-administered questionnaire (Ortega et al., 2015), in which participants documented all foods consumed each day, including ingredients used in meal preparation. They were also asked to indicate portion sizes using common household measures (e.g., cup, portion, glass, tablespoon, slice, handful, plate, or ladle) or the exact weight of the food or beverage. To support this process, participants were provided with a guide containing standard weight and volume conversions (Dapcich et al., 2004) [accessed 4 September 2021].

The Food Frequency Questionnaire (Trinidad Rodríguez et al., 2008) included a table where participants indicated how often they typically consumed each of the aforementioned food groups.

#### 2.9.2 Diet and eating habits assessment

Based on the seven-day dietary records and the Food Frequency Questionnaire data, the quality of participants’ diets was assessed using DietoPro®^1^, a nutritional management software developed in Valencia, Spain. This tool generates individualized nutritional profiles by calculating the daily averages of macronutrient and micronutrient intake.

For macronutrients, the software provided data on total energy intake, protein, carbohydrates, total fat, and a detailed lipid profile (including monounsaturated, saturated, and polyunsaturated fatty acids), cholesterol, and the percentage distributions of proteins, fats, and carbohydrates.

For micronutrients, daily intake levels were calculated for sodium, fiber, ethanol, iodine, potassium, calcium, magnesium, phosphorus, iron, selenium, zinc, vitamins B1, B2, B6, B12, folate, niacin, and vitamins C, A, D, and E. Additionally, composite nutritional indicators were assessed, such as the calcium/phosphorus, vitamin E/polyunsaturated fatty acids, and vitamin B6/protein ratios.

To assess whether participants’ diets met nutritional recommendations, we referred to the Dietary Reference Intakes (DRIs) established for the Spanish population, including the official guidelines from the “Dietary Reference Intakes for the Spanish Population” (Cuervo et al., 2010) and the consensus document by the Spanish Society of Community Nutrition (SENC) (Aranceta and Serra, 2011).

## 3 Results

Given the limited number of PEG patients (*n* = 5), all PEG-related analyses should be interpreted as exploratory and hypothesis-generating.

### 3.1 Demographic and clinical data

Table 1 presents the demographic and clinical data of the participants. Homogeneity testing was conducted to compare the demographic and clinical variables between patients with and without PEG. No statistically significant differences were found in age (*p* = 0.845) or time of diagnosis (*p* = 0.648) between the groups. Similarly, the sex distribution did not differ significantly (*p* = 0.114). However, a significant difference was observed in the distribution of ALS type (Bulbar vs. Spinal) between PEG and non-PEG patients (*p* = 0.013), suggesting a potential association between PEG status and ALS phenotype.

**Table 1:** Demographic data of patients.

Characteristics	ALS group (NO PEG)	PEG group
*N*	54	5
Age	57.11 ± 10.4	58 ± 11.77
Sex (% female)	33	80
Bulbar	12.96	80
Spinal	87.04	20
Time of diagnosis	31.67 ± 32.7	38 ± 19.92

### 3.2 Microbial taxonomic analysis

**Figure 1:**
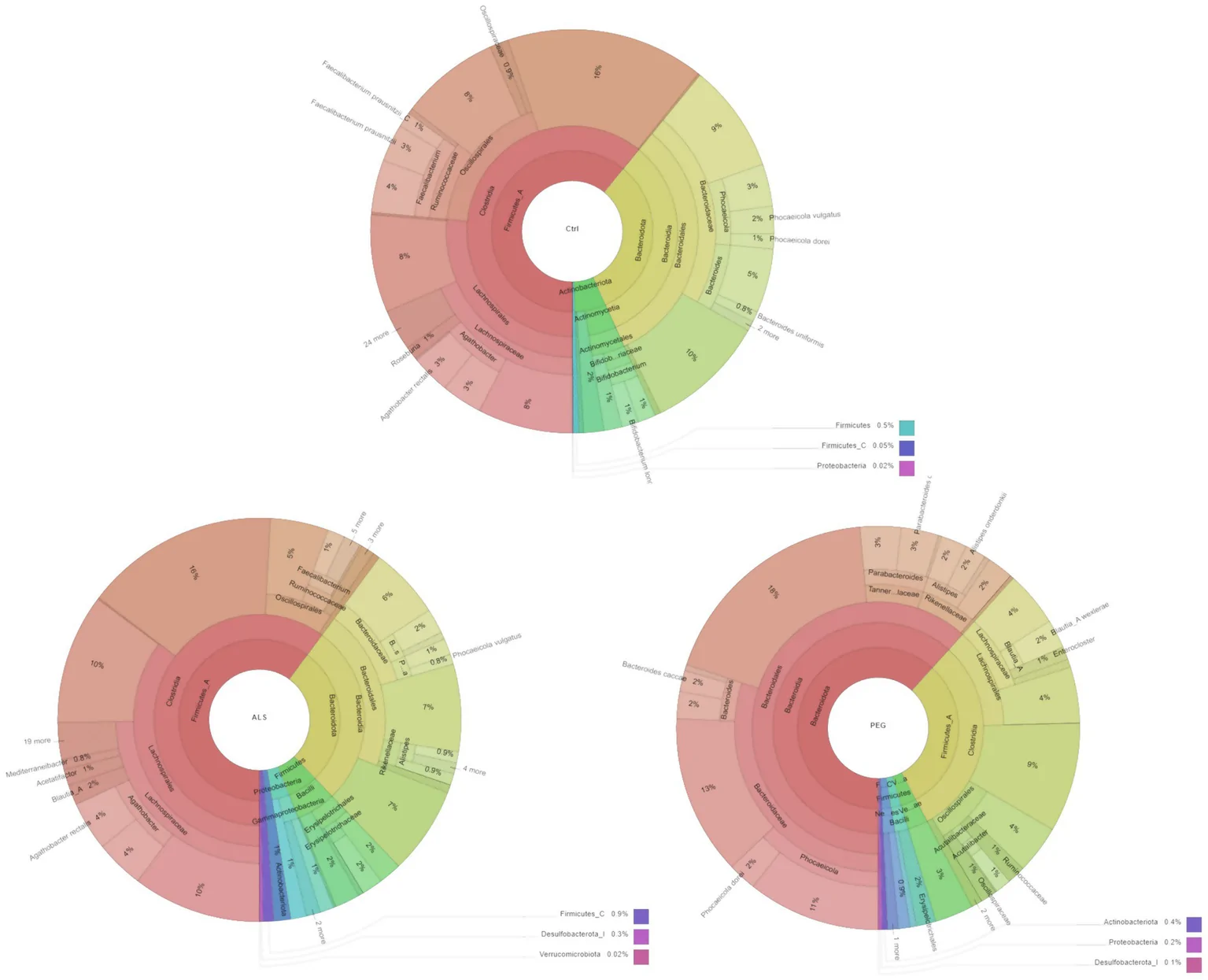
Krona plots display hierarchical taxonomic composition as nested radial sectors; the innermost ring represents domain level, with successive outer rings corresponding to progressively finer taxonomic ranks (phylum, class, order, family, genus, species). The angular size of each sector is proportional to the relative abundance of that taxon. Key features to note: the dominant position of Firmicutes_A-affiliated taxa across all groups, and the relatively greater representation of Bacteroidota and reduced Firmicutes_A in the PEG group **(c)** compared to controls **(a)** and ALS without PEG **(b)**.

Diversity analyses provided a detailed taxonomic overview of the samples, highlighting both the richness and complexity of the microbial communities present (Figure 1; pseudo-counts are reported in Supplementary file 1). Across all samples, a total of 2 domains, 17 phyla, 21 classes, 54 orders, 129 families, 645 genera and 1,580 species were identified (Supplementary file 5). After applying the prevalence filter (taxa present in ≥70% of samples in at least one group), 1 domain, 8 phyla, 9 classes, 15 orders, 30 families, 93 genera and 115 species were retained for downstream analyses (Supplementary file 6). The complete taxonomic lists are provided in Supplementary files 1, 2, and the taxonomic validity status of each taxon is reported in Supplementary file 2. Statistical analyses were performed across all detected taxa; however, only results corresponding to taxa meeting the prevalence criterion are reported and discussed below.

Taxonomic analyses were used to describe cross-sectional bacterial diversity differences among groups, and no temporal or causal inferences were drawn from these comparisons.

To ensure the robustness of the analysis, only taxa detected in at least 70% of the representatives of each group were considered. This filtering criterion allowed for a more consistent and reliable comparison across the different conditions studied, minimizing the impact of rare or sporadic taxa and focusing on the core microbiota shared within each group.

At the phylum level, microbial diversity was dominated by four major lineages: *Firmicutes_A* (a lineage within the GTDB taxonomy used by MetaPhlAn4, corresponding to a clade formerly classified within the traditional phylum Firmicutes but reclassified as a distinct lineage; the “_A” suffix denotes its GTDB designation), *Bacteroidota*, *Actinobacteriota* and *Proteobacteria*. *Firmicutes_A* was the most abundant phylum overall, with abundances of 54.50% in controls, 51.52% in ALS and 42.23% in PEG patients. *Bacteroidota* showed more homogeneous distribution across groups, with abundances of 34.72% in controls, 33.39% in ALS and 37.11% in PEG. *Actinobacteriota* was notably more abundant in PEG (7.80%) compared to ALS (4.82%) and controls (4.97%), and *Verrucomicrobiota* also showed a markedly higher presence in PEG (8.48%) compared to both ALS (1.01%) and controls (1.07%). *Proteobacteria* was more abundant in ALS (3.46%) than in PEG (1.08%) and controls (1.65%).

At the class level, *Clostridia* was the predominant class across all groups, comprising the bulk of *Firmicutes_A* diversity. At the genus level, notable differences were observed across groups. *Faecalibacterium* showed the most pronounced inter-group variation, with mean relative abundances of 2.50% in controls, 0.77% in ALS and 0.36% in PEG—a progressive reduction from controls to ALS to PEG. *Phocaeicola* showed the opposite trend, with higher abundance in PEG (1.90%) than in controls (1.36%) and ALS (1.30%). *Bacteroides* and *Alistipes* were relatively stable across groups.

At the species level, *Bifidobacterium longum* showed higher abundance in PEG (0.27%) compared to controls (0.22%) and ALS (0.13%). *Faecalibacterium prausnitzii*, widely recognized for its anti-inflammatory properties, was most abundant in controls (0.85%), followed by ALS (0.24%) and PEG (0.23%).

The Firmicutes/Bacteroidetes (F/B) ratio, although increasingly considered an oversimplification of gut microbiota composition, was calculated for descriptive purposes. Values were 0.327 in ALS patients, 0.310 in controls and 0.201 in PEG patients, indicating a Bacteroidetes-dominant microbiota across all three groups and a markedly more pronounced imbalance in PEG patients, whose ratio was notably lower than both ALS and controls, consistent with the broader compositional alterations observed in this subgroup.

### 3.3 Bacterial diversity

Alpha diversity analysis revealed statistically significant differences between groups for the Shannon index (Kruskal-Wallis: H = 16.40, *p* = 0.0003), Simpson index (H = 10.36, *p* = 0.006) and Pielou’s evenness (H = 9.46, *p* = 0.009), but not for observed richness, Chao1 or ACE (*p* > 0.1). Pairwise comparisons showed that ALS patients presented significantly higher Shannon diversity than controls (Mann–Whitney U, *p* = 0.001, *q* = 0.011) and higher than PEG patients (*p* = 0.007, *q* = 0.031). No significant differences were observed between controls and PEG (*p* = 0.791). Mean values were: Shannon (ALS = 3.690, PEG = 3.499, Ctrl = 3.537), Simpson (ALS = 0.934, PEG = 0.930, Ctrl = 0.929), Chao1 (ALS = 440.9, PEG = 359.8, Ctrl = 400.1) and observed richness (ALS = 434.1, PEG = 353.7, Ctrl = 395.5).

Beta diversity analysis based on Aitchison distance revealed statistically significant differences in overall community composition between groups (PERMANOVA: *F* = 1.421, *R*^2^ = 0.036, *p* = 0.015), indicating that, despite similar alpha diversity, the three groups differ in their microbial community structure. However, betadisper analysis also showed significant differences in multivariate dispersion between groups (*F* = 8.516, *p* = 0.002), indicating that part of the compositional differences detected by PERMANOVA may reflect heterogeneity in within-group variability rather than a shift in group centroids alone. These results should therefore be interpreted with caution.

The statistical results of alpha and beta diversity analyses, including global tests, pairwise comparisons and group-wise mean values, are provided in Supplementary file 4.

### 3.4 Genus-level analysis

At the genus level, the most abundant taxa across all samples were *Firmicutes_A*-affiliated genera, consistent with the phylum-level results. *Faecalibacterium* was the most abundant genus overall (mean relative abundance across all samples: 7.46%), followed by *Blautia_A* (5.97%), *Bacteroides* (4.48%), *Alistipes* (4.48%) and *Dysosmobacter* (4.48%). These were followed by *Acetatifactor*, *Lawsonibacter*, *Mediterraneibacter*, *Choladocola*, *Roseburia*, *Phocaeicola*, *Agathobacter*, *Gemmiger*, *Ruthenibacterium* and *Enterocloster*, each contributing approximately 2.99% to the total community. The marked reduction of *Faecalibacterium* from controls to ALS to PEG, alongside the relative enrichment of *Phocaeicola* and *Bifidobacterium* in PEG patients, suggests group-specific shifts in the core microbiota that are further explored in the differential abundance analyses below.

### 3.5 Correlation between microbiota and PEG condition

The statistical study of MetaPhlAn’s taxonomic abundance (Supplementary file 2) for Ctrl, PEG and ALS included the mean, median, standard deviation, and coefficient of variation. The results of hypothesis testing and correlation analyses highlighted significant differences and correlations with ALS disease. Several taxa showed significant differences in the PEG group compared to the ALS and Control groups. These findings highlight significant alterations in the gut microbiota of patients with PEG, suggesting potential associations between specific bacterial taxa and their clinical conditions. Further research is required to elucidate the functional implications of these microbiota changes on disease management. The validity status of the samples was confirmed, ensuring reliable data for comparative studies.

#### 3.5.1 Taxonomical correlation with ALS group

Correlation analyses between taxon abundance in control and ALS group identified 12 taxa with statistically significant Spearman correlations (*p* < 0.05), though all associations were modest in magnitude, with absolute correlation coefficients ranging from 0.26 to 0.35. Among the positive associations, *Bacilli* (ALS = 0.78%, *r* = 0.331, *p* = 0.011), *Erysipelotrichales* (ALS = 0.32%, *r* = 0.301, *p* = 0.021), *Agathobacter rectalis* (ALS = 0.36%, *r* = 0.300, p = 0.021), *Agathobacter* (ALS = 0.58%, *r* = 0.290, *p* = 0.026), *Anaerovoracaceae* (ALS = 0.08%, *r* = 0.278, *p* = 0.033) and *Faecalibacterium* (ALS = 0.77%, *r* = 0.257, *p* = 0.050) showed higher abundance in ALS. On the negative side, *Butyricimonas* (ALS = 0.04%, *r* = −0.345, *p* = 0.007), *Bacteroidales* (ALS = 5.53%, *r* = −0.288, *p* = 0.027) and *Bacteroidia* (ALS = 5.53%, *r* = −0.288, *p* = 0.027) presented the strongest inverse associations. These findings should be interpreted with caution and require validation in larger cohorts.

#### 3.5.2 Taxonomical correlation with PEG subgroup

Given the limited sample size of the PEG subgroup (*n* = 5), the following results are strictly exploratory and should be interpreted with considerable caution. With this caveat in mind, 14 taxa showed statistically significant Spearman correlations with total microbial load (estimated total counts per sample) (*p* < 0.05), identified across both ALS vs. PEG and Controls vs. PEG comparisons. All associations displayed strong correlation coefficients (|*r*| ≥ 0.83), which is not uncommon in very small samples and may partly reflect statistical artefacts rather than true biological signal. The majority were negative, with *Christensenellaceae* (PEG = 0.01%, *r* = −0.943, *p* = 0.005), *Anaerobutyricum soehngenii* (PEG = 0.05%, *r* = −0.928, *p* = 0.008) and *Coprococcus_A* (PEG = 0.04%, *r* = −0.899, *p* = 0.015) showing the most pronounced inverse associations. *Dorea_A* (PEG = 0.13%, *r* = −0.886, *p* = 0.019), *Acetatifactor* (PEG = 0.07%, *r* = −0.841, *p* = 0.036), *Bifidobacterium* (PEG = 0.66%, *r* = −0.845, *p* = 0.034) and *Faecalibacterium prausnitzii* (PEG = 0.23%, *r* = −0.829, *p* = 0.042) also showed significant negative associations. In the opposite direction, *Parabacteroides* (PEG = 0.55%, *r* = 0.886, *p* = 0.019) and *Bacteroides caccae* (PEG = 0.22%, *r* = 0.886, *p* = 0.019) presented positive correlations with total microbial load. These observations may point to potentially interesting trends worthy of further investigation in adequately powered studies. Importantly, with *n* = 5, Spearman coefficients above |0.83| are mathematically near-inevitable and a single data point determines the entire result. No correction for multiple testing was applied across the many taxon-load pairs tested; as a consequence, these analyses must be treated exclusively as hypothesis generation and cannot be interpreted as even suggestive evidence of a true biological association (see Table 2).

**Table 2:** *Firmicutes* and *Bacteroidetes* abundance and ratios.

Group	*Firmicutes* (mean)	*Bacteroidetes* (mean)	F/B ratio
ALS	127686.263	390753.034	0.327
PEG	6832.552	33957.361	0.201
Ctrl	31313.258	101127.765	0.310

### 3.6 Differential abundance analysis between groups

The complete results of the differential diversity analyses are provided in Supplementary file 2. Taxa were considered differentially abundant when identified as significant by at least two of the three methods applied (ANCOMBC2, ALDEx2 and CLR-LM). As described in section 3.2, only taxa meeting the prevalence criterion (detected in ≥70% of samples in at least one group) are reported.

Note that all comparisons involving the external control group (3.6.2 and 3.6.3) are subject to potential technical batch effects, and should be considered with additional caution relative to the fully internal ALS vs. PEG comparison (3.6.1).

#### 3.6.1 ALS vs. PEG

Thirty-four taxa were significantly differentially abundant between ALS and PEG patients. The most robust findings—significant across all three methods—included the enrichment of *Coprobacter* (ALS = 0.01%, PEG = 0.07%) and *Coprobacter fastidiosus* (ALS = 0.01%, PEG = 0.05%) in PEG relative to ALS, alongside *Fimisoma* (ALS = 0.02%, PEG = 0.10%) and *Anaerotruncus massiliensis* (ALS = 0.003%, PEG = 0.02%). Among taxa significantly depleted in PEG, *Agathobacter* (ALS = 0.58%, PEG = 0.07%), *Agathobacter rectalis* (ALS = 0.36%, PEG = 0.06%), *Lachnospira* (ALS = 0.23%, PEG = 0.02%), *Roseburia intestinalis* (ALS = 0.10%, PEG = 0.00%), *Roseburia inulinivorans* (ALS = 0.10%, PEG = 0.01%), *Faecalibacterium prausnitzii_D* (ALS = 0.13%, PEG = 0.01%), *Faecalibacterium prausnitzii_G* (ALS = 0.14%, PEG = 0.00%) and *Gemmiger formicilis* (ALS = 0.13%, PEG = 0.01%) were consistently reduced in PEG, pointing to a broad depletion of butyrate-producing taxa.

#### 3.6.2 ALS vs. controls

Thirty-five taxa were significantly differentially abundant between ALS and control groups. The most notable finding was the consistent and significant reduction of *Faecalibacterium* in ALS patients relative to controls (ALS = 0.77%, Ctrl = 2.50%), alongside *Oscillospirales* (ALS = 5.03%, Ctrl = 7.56%), *Lawsonibacter asaccharolyticus* (ALS = 0.02%, Ctrl = 0.06%), *Faecalibacterium prausnitzii* (ALS = 0.24%, Ctrl = 0.85%), *Gemmiger qucibialis* (ALS = 0.08%, Ctrl = 0.26%), *Bifidobacterium* (ALS = 0.35%, Ctrl = 0.48%) and *Bifidobacterium longum* (ALS = 0.13%, Ctrl = 0.22%). Conversely, *Anaerobutyricum soehngenii* (ALS = 0.05%, Ctrl = 0.00%), *Bacilli* (ALS = 0.78%, Ctrl = 0.34%), *Erysipelotrichales* (ALS = 0.32%, Ctrl = 0.11%), *Peptostreptococcales* (ALS = 0.14%, Ctrl = 0.05%), *Anaerovoracaceae* (ALS = 0.08%, Ctrl = 0.04%), *Odoribacter* (ALS = 0.13%, Ctrl = 0.04%), *Coprobacillaceae* (ALS = 0.13%, Ctrl = 0.05%) and *Erysipelotrichaceae* (ALS = 0.20%, Ctrl = 0.06%) were significantly more abundant in ALS, suggesting an alteration in the composition of the gut microbiota that warrants further investigation.

#### 3.6.3 Controls vs. PEG

Forty-five taxa were significantly differentially abundant between controls and PEG patients. The most striking finding was the marked and significant reduction of *Faecalibacterium* in PEG (Ctrl = 2.50%, PEG = 0.36%), consistent across all three methods, together with *Gemmiger qucibialis* (Ctrl = 0.26%, PEG = 0.02%), *Agathobacter* (Ctrl = 0.83%, PEG = 0.07%), *Agathobacter rectalis* (Ctrl = 0.74%, PEG = 0.06%), *Acetatifactor* (Ctrl = 0.22%, PEG = 0.07%), *Faecalibacterium prausnitzii_D* (Ctrl = 0.31%, PEG = 0.01%), *Faecalibacterium prausnitzii_G* (Ctrl = 0.32%, PEG = 0.00%), *Roseburia intestinalis* (Ctrl = 0.04%, PEG = 0.00%) and *Eisenbergiella* (Ctrl = 0.06%, PEG = 0.01%). Conversely, *Actinomycetia* (Ctrl = 0.48%, PEG = 0.66%), *Fimisoma* (Ctrl = 0.01%, PEG = 0.10%), *Anaerotruncus massiliensis* (Ctrl = 0.00%, PEG = 0.02%), *Peptostreptococcales* (Ctrl = 0.05%, PEG = 0.14%), *Anaerovoracaceae* (Ctrl = 0.04%, PEG = 0.13%) and *Bifidobacterium* (Ctrl = 0.48%, PEG = 0.66%) were significantly enriched in PEG relative to controls. The progressive compositional disruption observed from controls to ALS to PEG suggests a continuum of gut microbial deterioration, potentially exacerbated in PEG patients by enteral feeding and advanced disease (Rowin et al., 2017). The consistent depletion of Firmicutes-affiliated butyrate producers across comparisons further supports the picture of impaired SCFA production capacity in these patients (Louis and Flint, 2017).

### 3.7 Nutrition and taxonomy

Significant Spearman correlations were identified between dietary nutrient intake and specific taxa in PEG patients (Supplementary file 3). Calcium showed strong positive correlations with *Dorea formicigenerans* (*r* = 0.97) and *Lacrimispora* (*r* = 0.98). Cholesterol was positively correlated with *Anaerobutyricum hallii* (*r* = 0.98). Ethanol showed negative correlations with *Hungatella* and *Hungatella hathewayi* (both *r* = −0.97), while *Roseburia* showed a positive correlation (*r* = 0.98). Folate and phosphorus showed highly similar correlation patterns, both positively correlating with *Prevotellaceae* (*r* = 0.99), *Gemmiger formicilis*, *Anaerobutyricum hallii*, *Blautia massiliensis*, *Lachnospira*, *Lachnospira eligens* and *Ruminococcus* (all *r* = 1.00). Iron, niacin, vitamin B12 and zinc showed negative correlations with *Hungatella* and *Hungatella hathewayi* (*r* ranging from −0.97 to −1.00) and positive correlations with *Roseburia* (*r* ranging from 0.98 to 0.99). Potassium correlated positively with *Prevotellaceae* (*r* = 0.98), *Candidatus Allochristensenella* (*r* = 1.00), *Gemmiger formicilis* (*r* = 0.98), *Anaerobutyricum hallii* (*r* = 0.97), *Blautia massiliensis*, *Lachnospira*, *Lachnospira eligens* and *Ruminococcus* (all *r* = 0.98). Selenium was positively correlated with *Alistipes ihumii* and *Roseburia* (both *r* = 0.98). Vitamin B6 correlated with *Prevotellaceae* (*r* = 0.96) and *Alistipes ihumii* (*r* = 0.99). Vitamin D showed negative correlations with *Eggerthella* and *Gordonibacter* (both *r* ≈ −1.00). Vitamin E correlated positively with *Firmicutes* (*r* = 0.99) and *Enterobacterales* (*r* = 1.00). Given the small sample size of the PEG subgroup (*n* = 5), the reported correlations should be regarded as exploratory. These results cannot be interpreted as suggestive evidence of any biological association and are presented solely as hypothesis generation for future adequately powered studies.

## 4 Discussion

The gut microbiota has emerged as a relevant factor in the pathogenesis and progression of neurodegenerative diseases, including ALS, through its bidirectional communication with the central nervous system via the gut-brain axis (Boddy et al., 2021; Blacher et al., 2019; Fang et al., 2016; Gautam et al., 2025b). The present study identified compositional alterations in the gut microbiota of ALS patients, with the most extensive changes observed in those requiring PEG, suggesting a continuum of microbial disruption associated with disease severity and nutritional status. However, before discussing the specific findings, an important methodological consideration should be noted. The control group was obtained from an external public dataset (BioProject PRJNA961076) rather than being recruited and processed together with the patient cohort. As a result, differences in sample collection, DNA extraction procedures, sequencing runs, and bioinformatic processing may have introduced batch effects that cannot be completely excluded.

Therefore, observations involving comparisons between controls and patients, including the apparent progressive reduction of SCFA-producing bacteria from controls to ALS and PEG patients, should be interpreted with caution. In contrast, the comparison between ALS and PEG patients was performed using samples processed under the same experimental and analytical conditions, making this analysis methodologically more robust and, consequently, the most reliable result of the study.

### 4.1 Correlations with ALS

Among the taxa with sufficient representation in the cohort, 12 showed statistically significant Spearman correlations with ALS group, although all associations were modest in magnitude (|*r*| ≤ 0.35). *Butyricimonas* showed the strongest inverse correlation (*r* = −0.345, *p* = 0.007), suggesting that higher abundance of this genus may be associated with ALS. Reduced *Butyricimonas* abundance has been previously reported in ALS patients across independent cohorts and associated with neurodegeneration, potentially through its role in SCFA production and anti-inflammatory gut-brain axis signaling (Cuevas-Sierra et al., 2024). *Bacteroidales* and *Bacteroidia* also showed significant inverse associations, suggesting that higher abundance of these Bacteroidetes lineages may be associated with ALS disease, although the biological basis of this association remains unclear and should be interpreted with caution given the cross-sectional nature of the study.

Positive correlations with ALS group were observed for *Bacilli* (*r* = 0.331, *p* = 0.011), *Erysipelotrichales* (*r* = 0.301, *p* = 0.021), *Agathobacter rectalis* (*r* = 0.300, *p* = 0.021), *Agathobacter* (*r* = 0.290, *p* = 0.026), *Anaerovoracaceae* (*r* = 0.278, *p* = 0.033) and *Faecalibacterium* (*r* = 0.257, *p* = 0.050). The positive correlation of *Agathobacter rectalis* with ALS group is particularly noteworthy given that this butyrate-producing member of the *Lachnospiraceae* family has been previously associated with neuroprotective effects in Alzheimer’s disease models through butyrate-mediated suppression of microglial activation via the Akt/NF-κB pathway (Lv et al., 2024). This apparent paradox—a potentially neuroprotective taxon correlating positively with severity—may reflect a reactive increase in response to neuroinflammation, a compensatory mechanism, or confounding by disease-related dietary changes, and warrants careful interpretation and further investigation. Similarly, the positive correlation of *Faecalibacterium* with ALS severity in this comparison contrasts with its well-established anti-inflammatory role and its depletion in the differential abundance analyses—a discrepancy that underscores the complexity of cross-sectional correlational analyses in small cohorts. Given the modest effect sizes observed across all associations, these findings should be considered exploratory and require confirmation in larger longitudinal cohorts.

### 4.2 Correlations in the PEG subgroup

In the PEG subgroup (*n* = 5), 14 taxa showed statistically significant Spearman correlations with ALS group, with all associations displaying very strong coefficients (|*r*| ≥ 0.83). These results must be interpreted with considerable caution, as such strong correlations are mathematically expected in very small samples and may reflect statistical artefacts rather than true biological signal. *Christensenellaceae* showed the strongest inverse correlation (*r* = −0.943, *p* = 0.005). This family is one of the most heritable in the human gut microbiome and has been consistently associated with favorable metabolic and inflammatory profiles across multiple disease contexts (Stojanov et al., 2020; Waters and Ley, 2019). *Anaerobutyricum soehngenii* (*r* = −0.928, *p* = 0.008) is a propionate and butyrate producer whose depletion has been associated with gut barrier dysfunction (Shetty et al., 2018). *Coprococcus_A* (*r* = −0.899, *p* = 0.015), *Dorea_A* (*r* = −0.886, *p* = 0.019), *Acetatifactor* (*r* = −0.841, *p* = 0.036), *Bifidobacterium* (*r* = −0.845, *p* = 0.034) and *Faecalibacterium prausnitzii* (*r* = −0.829, *p* = 0.042) were also inversely associated with PEG group, pointing collectively to a depletion of anti-inflammatory and SCFA-producing bacteria with worsening clinical status (Zhai et al., 2019). Conversely, *Parabacteroides* (*r* = 0.886, *p* = 0.019) and *Bacteroides caccae* (*r* = 0.886, *p* = 0.019) showed positive correlations, suggesting enrichment of these Bacteroidetes taxa with greater severity. These observations may point to potentially interesting trends worthy of further investigation in adequately powered studies.

### 4.3 Differential abundance analysis

The most consistent finding across differential abundance analyses was the significant depletion of *Faecalibacterium*, and particularly *Faecalibacterium prausnitzii*, in both ALS and PEG patients. *Faecalibacterium prausnitzii* is one of the most abundant and functionally active members of the human gut microbiome, widely recognized for its anti-inflammatory properties and its role as a major butyrate producer (Sokol et al., 2008; Miquel et al., 2013). Its depletion has been consistently reported in inflammatory and neurodegenerative conditions (Boddy et al., 2021; Zhang et al., 2022; Gautam et al., 2025a). The progressive reduction from controls (2.50%) to ALS (0.77%) to PEG (0.36%) observed here reinforces the hypothesis that *Faecalibacterium* depletion may be both a marker and a potential contributor to ALS disease. Consistent with this, *Gemmiger qucibialis*—another butyrate-associated commensal—was also significantly reduced in both ALS and PEG.

*Agathobacter* and *Agathobacter rectalis* were significantly depleted in PEG relative to both ALS and controls. As noted above, *Agathobacter rectalis* produces butyrate and has demonstrated neuroprotective effects in neurodegeneration models (Lv et al., 2024), and its reduced abundance in PEG patients further supports the picture of impaired butyrate-producing capacity. Similarly, *Lachnospira*, *Roseburia intestinalis*, *Roseburia inulinivorans*, *Gemmiger formicilis* and *Faecalibacterium prausnitzii_D* and *_G* strains were all significantly depleted in PEG relative to ALS, pointing to a broad reduction of the SCFA-producing Lachnospiraceae community in the most severely affected patients (Louis and Flint, 2017). *Acetatifactor* and *Eisenbergiella* were additionally depleted in PEG relative to controls.

In the ALS versus controls comparison, beyond the reduction of *Faecalibacterium* and *Oscillospirales* (ALS = 5.03%, Ctrl = 7.56%), *Lawsonibacter asaccharolyticus*, *Gemmiger qucibialis*, *Bifidobacterium* (ALS = 0.35%, Ctrl = 0.48%) and *Bifidobacterium longum* (ALS = 0.13%, Ctrl = 0.22%) were also significantly reduced in ALS. The reduction of *Bifidobacterium* in ALS is consistent with recent metagenomic evidence reporting alterations in *Bifidobacterium* prevalence in ALS individuals (Gautam et al., 2025a). Conversely, *Anaerobutyricum soehngenii* (ALS = 0.05%, Ctrl = 0.00%), *Odoribacter*, *Bacilli*, *Erysipelotrichales*, *Peptostreptococcales*, *Anaerovoracaceae*, *Coprobacillaceae* and *Erysipelotrichaceae* were significantly more abundant in ALS. The functional implications of these shifts warrant further investigation.

*Fimisoma*, *Coprobacter* and *Coprobacter fastidiosus* were significantly enriched in PEG relative to both ALS and controls, alongside *Anaerotruncus massiliensis*, *Actinomycetia* and *Massilimaliae timonensis*. The functional roles of these taxa in the context of ALS and PEG remain poorly characterized and warrant further investigation. The progressive compositional disruption observed from controls to ALS to PEG suggests a continuum of gut microbial deterioration, potentially exacerbated in PEG patients by enteral feeding and advanced disease (Rowin et al., 2017). The consistent depletion of Firmicutes-affiliated butyrate producers across comparisons further supports the picture of impaired SCFA production capacity in these patients (Louis and Flint, 2017).

### 4.4 Associations between nutrient intake and gut microbiota in PEG patients

The nutrient-microbiota correlations observed in PEG patients reveal patterns that are biologically coherent despite the exploratory nature of the findings. The consistent positive associations between multiple micronutrients—particularly folate, potassium, phosphorus and calcium—and members of the *Lachnospiraceae* family (*Anaerobutyricum hallii*, *Blautia massiliensis*, *Lachnospira*, *Gemmiger formicilis*) suggest that higher nutrient intake in PEG patients may support the growth of SCFA-producing taxa, reinforcing the link between nutritional adequacy and gut microbiota composition (Louis and Flint, 2017; Makki et al., 2018). *Anaerobutyricum hallii* is a butyrate and propionate producer whose adequate intake has been linked to gut barrier integrity and anti-inflammatory signaling (Shetty et al., 2018). The negative correlations of iron, niacin, vitamin B12, zinc and ethanol with *Hungatella*—and their simultaneous positive association with *Roseburia*—are particularly consistent, suggesting a possible nutrient-driven competitive dynamic between these two genera. *Roseburia* is a well-characterized butyrate producer (Tamanai-Shacoori et al., 2017), while *Hungatella* has been less studied in this context. The near-perfect correlation of vitamin D with negative abundance of *Eggerthella* and *Gordonibacter* is remarkable considering the established role of vitamin D in gut immune regulation and its reported association with microbiome composition in neurological disease (Di Somma et al., 2017). Overall, these findings suggest that the nutritional profile delivered through PEG may partially modulate gut microbiota composition, and that optimizing nutrient intake in this population could represent a strategy to preserve beneficial taxa. However, the very small sample size precludes any definitive conclusions, and these observations should be validated in larger studies.

## 5 Limitations

This study had several limitations that should be acknowledged. The cross-sectional design precludes the establishment of causal relationships between microbiota alterations and ALS group, limiting our understanding of the temporal dynamics of gut-brain axis dysfunction in ALS. Sample size constraints, particularly in the PEG subgroup (*n* = 5), substantially reduced statistical power and the exploratory correlation analyses reported for this subgroup should be interpreted with considerable caution. The selective sampling approach may introduce selection bias and limit generalizability across diverse ALS populations and geographic regions.

Control samples were obtained from a publicly available external dataset (BioProject PRJNA961076), which may introduce batch effects due to differences in sample collection, DNA extraction protocols, sequencing platforms and bioinformatic processing relative to the ALS and PEG cohorts. Although normalization procedures were applied to minimize these effects, residual technical variability cannot be excluded and may have influenced the observed compositional differences between groups. This represents a major limitation of the study and should be considered when interpreting between-group comparisons involving the control group.

The reliance on shotgun sequencing provides limited functional insight into metabolic pathways and microbial activity, as it captures only the presence of genes rather than their active expression. Additionally, the absence of longitudinal data prevents the assessment of microbiota stability over disease progression, and potential confounding variables such as antibiotic use, BMI, disease duration, ALSFRS-R score, medication history, dietary patterns prior to symptom onset and environmental exposuresshould be considered in future studies.

In this sense, future investigations should prioritize longitudinal cohort studies to elucidate the temporal relationship between changes in the microbiota and the progression of neurodegeneration. Larger multicenter studies incorporating standardized clinical assessments and comprehensive metabolomic profiling would strengthen the evidence base. Functional metagenomics and transcriptomics approaches should be employed to characterize microbial metabolic capacity and active pathways, particularly focusing on SCFA production, neurotransmitter synthesis and inflammatory mediator generation. Research efforts should also explore targeted therapeutic interventions, including precision probiotic formulations designed to restore the beneficial taxa identified in this study, prebiotic strategies to enhance SCFA-producing bacteria, and comprehensive nutritional interventions tailored to individual microbiome profiles. Finally, the development of microbiome-based biomarkers for disease monitoring and treatment response prediction represents a promising avenue for personalized ALS management.

## 6 Conclusion

This study revealed distinct gut microbiota alterations in patients with ALS, with notable differences between those requiring PEG and those maintaining oral nutrition. The observed taxonomic shifts, characterized by reduced Firmicutes/Bacteroidetes ratios in ALS-PEG patients and the differential abundance of key metabolically active genera, suggest a complex interplay between nutritional status, feeding route, and microbial ecosystem dysfunction. Specific correlations between bacterial taxa and ALS and PEG groups suggest potentially interesting associations, though these should be interpreted with caution given the cross-sectional design and the limited sample sizes involved. The identification of alterations in SCFA-producing bacteria, particularly involving *Faecalibacterium*, *Butyricimonas*, and *Lachnospiraceae* family members, supports the hypothesis that metabolic dysfunction within the gut-brain axis contributes to ALS pathophysiology. These findings provide a foundation for developing microbiome-targeted therapeutic strategies and highlight the clinical relevance of gastrointestinal health in the management of ALS. Further research integrating functional analysis with longitudinal clinical assessments will be essential to translate these observations into actionable interventions for improving patient outcomes.

## Data Availability

The metagenomic sequencing data presented in the study are deposited in the European Nucleotide Archive (ENA) repository, accession number PRJEB90512. Repository names and accession numbers for any additional data are provided in the article and its Supplementary material.

## Footnotes

1
https://dietopro.com/
